# Digital inclusive finance, rural revitalization and rural consumption

**DOI:** 10.1371/journal.pone.0310064

**Published:** 2025-01-03

**Authors:** Wei Li, Lu Zhang, Mingyue Pu, Hui Wang

**Affiliations:** 1 Institute of Applied Economy, Shanghai Academy of Social Sciences, Shanghai, China; 2 School of Accounting and Finance, Anhui Xinhua University, Anhui, China; 3 Taiwan Research Institute, Xiamen University, Fujian, China; Universidad Mayor, CHILE

## Abstract

With the rapid development of technology and the evolution of the global financial system, digital inclusive finance has become a new way to promote rural revitalization and rural residents’ consumption with the power of financial technology. This study explores the relationship between digitally inclusive finance, rural revitalization, and rural residents’ consumption. Based on the panel data of 30 provinces and cities in China from 2011 to 2021, this study empirically examines the impact of digitally inclusive finance on rural residents’ consumption and the mediating and threshold effects of rural revitalization. The results reveal that digitally inclusive finance is conducive to the enhancement of rural residents’ consumption, and rural revitalization acts as a mediator. Meanwhile, there is a nonlinear positive correlation between the impact of digitally inclusive finance and rural revitalization on rural residents’ consumption, in which there is a double-threshold effect of digitally inclusive finance and rural revitalization in the lagged period. Based on the above findings, we believe that while promoting digitally inclusive finance, it is important to promote rural revitalization strategy in a timely manner, improve rural infrastructure, and continuously stimulate the impact of digitally inclusive finance on rural residents’ consumption.

## 1. Introduction

With the strategy of rural revitalization being incorporated into the overall development of the China, the comprehensive economic and social development of rural areas has become a key direction for future development. Further promotion and deepening of the strategy of rural revitalization will improve the infrastructure of the majority of rural areas and steadily increase the income of residents. The 2023 Central Economic Work Conference proposed stimulating current domestic consumption and further expanding domestic demand. However, the development of the vast rural areas is lagging, and most residents generally have a habit of saving. Based on the current stage of the country’s development, the 20th National Congress of the Communist Party of China (CPC) further emphasized the goal of modernization for the common prosperity of all people, coordinating urban and rural development, and realizing the goal of modern living conditions in the countryside. Consequently, it is essential to determine the role of financial resources in promoting rural modernization and achieving economic development and higher incomes for people in rural areas.

Through the rapid development of the Internet and the continuous improvement of digital infrastructure, the digital economy is becoming a new engine and kinetic energy source for the stable and high-quality development of China’s economy [1]. The predicament of the difficulty and high cost of financing for agriculture-related small, medium, and micro enterprises (SMEs) is widespread. The source of financing relies mainly on credit from banks, which cannot meet the demand for personalized financial products of SMEs. With the development of the Internet, blockchain, and other technologies and the development of digital infrastructure in rural areas, especially the deep integration and development of inclusive finance and fintech, the information asymmetry problem between the financing parties has been reduced to a greater extent [2]. In fact, financing constraints have been eased [3,4], which in turn accurately identifies the financing constraints [3,4], and thus accurate identification of financing subjects and stronger risk control [5,6], which not only meets the financial needs of agriculture-related SMEs for expanding reproduction, but also improves the employment and income level in rural areas.

The prosperous development of rural areas is closely related to the optimization of the rural industrial structure and the improvement in the income level of rural residents [7]. China’s long-term unbalanced development of urban and rural areas has led to the outflow of labor, raw materials, and other factors of production from rural areas, hindering the areas’ development, which in turn limits the growth of rural residents’ income and widens the development gap between urban and rural areas. As the global economic growth rate declines and the impact of epidemics increases, China is facing problems such as blocked exports, a surplus of domestic products, and a shortage of consumer demand. In the context of rural revitalization, how to promote consumption demand in rural areas has become an important issue, especially in terms of building a “double-cycle” development pattern. Subsequently, how to use the domestic macro-cycle to stimulate consumption demand in rural areas deserves in-depth consideration.

This study considers rural revitalization and rural residents’ consumption as the research objects and first adopts the fixed effect regression method and carries out the mediation effect test. In addition, it constructs a threshold model to empirically analyze the threshold effect between digitally inclusive finance, rural revitalization, and rural residents’ consumption. Through these methods, this study aims to explore the relationship between rural revitalization and rural residents’ consumption as well as the role of digitally inclusive finance in promoting rural residents’ consumption demand. The findings of this study will provide policymakers and relevant institutions with a basis for decision-making regarding rural revitalization and will also provide useful references for expanding rural residents’ consumption, optimizing the structure of rural industries, and raising the level of residents’ income.

The main contributions of this study are as follows: First, the relationship between digitally inclusive finance, rural revitalization, and rural residents’ consumption is examined from both linear and non-linear perspectives. Second, it extends the extant literature by examining not only digitally inclusive finance and rural revitalization as explanatory variables but also rural revitalization as a mediating variable to better clarify the relationship between the three factors.

## 2. Theoretical analysis and research hypothesis

### 2.1 Rural revitalization and rural consumption

The concept of rural revitalization was first proposed in 2017, and Xi Jinping proposed the comprehensive promotion of rural revitalization in a report of the 20th National Congress in 2022, emphasizing the solid promotion of rural industry, talent, culture, ecology, and organizational revitalization. At present, academic research on the rural revitalization of rural residents’ consumption is relatively limited, but some scholars have studied the impact of rural residents’ consumption from the dimension of rural revitalization. [8–12] analyzed the development of rural e-commerce from the perspective of the development of rural e-commerce, and concluded that improvement of rural infrastructure and development of rural e-commerce can increase the income of rural residents and promote the consumption and upgradation of rural residents. With regard to the industrial structure, [13] examined the shift in agricultural production from low-value food crops to high-value commodities in Indonesia over the past three decades and determined that the development of high-value agriculture was positively correlated with rural household income, which in turn improves the demand for life. [14,15] argued that optimizing the industrial structure is needed to achieve quality, expansion, and upgradation of rural consumption. In terms of financial support for agriculture, [16] analyzed the impact of government expenditure in a general equilibrium monopolistic competition model and found that an increase in government expenditure has a positive effect on residents’ consumption in both the short and long term. [17] found that financial support for agriculture has a certain effect on the consumption level of rural residents: with the positive impact of financial support for agriculture policy, the consumption level of rural residents will appear at a certain level of enhancement and shows a lag phenomenon; with the gradual prolongation of time, the impact gradually tends to level off. [18] analyzed the impact of financial support for agriculture on the consumption of rural residents from the level of consumption, consumption structure, and quality of consumption and found that the financial support for agricultural expenditure and the level of rural residents’ consumption has a segmented non-linear relationship, and the upgradation of the consumption structure of rural residents has a “U”-shaped non-linear relationship. With regard to the allocation of financial resources, [19,20] argued that credit constraints make consumers too sensitive to current consumption, which hinders the growth of their consumption. The realization of financial marketization can optimize the allocation of financial resources to facilitate consumers to make better use of financial resources to realize intertemporal consumption, thus effectively releasing the demand for residential consumption. Based on the above analyses, this study proposes the following:

Hypothesis 1: Rural revitalization effectively promotes rural residents’ consumption.

### 2.2 Digitally inclusive finance and rural revitalization

The strategy of rural revitalization was proposed by Comrade Xi Jinping in the report of the 19th Party Congress on October 18, 2017, emphasizing the solution for the “three rural issues,” which mainly involves five dimensions: industrial prosperity, ecological livability, civilized countryside, effective governance, and affluent life. [21,22] used these five dimensions and measured the indicators of rural revitalization to construct indicators. With the development of financial technology and the promotion of rural revitalization strategies, scholars are first studying rural revitalization from the perspective of digitally inclusive finance. [23,24] found that digitally inclusive finance, with its technological advantages, enhances credit volume, achieves surface expansion, and ensures quality improvement, promotes the development of rural industries, and helps rural industrial revitalization. In terms of improving digital infrastructure, [25,26] found that digital financial inclusion significantly contributes toward the development level of rural revitalization, there is significant regional heterogeneity, and digital infrastructure construction and rural human capital significantly moderate the relationship between digitally inclusive finance and the development of rural revitalization. With regard to financing costs, [27,28] argued that the combination of digitally inclusive finance classes with modern technology can realize the cost effect and, therefore, meet the demand for financing rural industrial development. [29,30] believd that digitally inclusive finance, as an emerging product of the financial industry, can broaden financing channels and improve financing efficiency, compensate for defects in expensive traditional financial services, and effectively provide reliable financial support and convenient services for the innovative and entrepreneurial activities of various financing subjects. Based on the above analyses, this study proposes the following hypothesis:

Hypothesis 2: Digitally inclusive finance can effectively promote rural revitalization.

Scholars have begun to conduct research on the five dimensions of digital financial inclusion for rural revitalization. In terms of industrial prosperity, [31] argued that digitally inclusive finance can directly affect rural industrial prosperity through the breadth of coverage, diversification of financial products, and low cost, and indirectly support rural industrial prosperity by supporting the regional economy. [32] found that digitally inclusive finance provides good support for the development of rural industrial integration by increasing capital supply, driving technological innovation, and guiding the return of labor. [33] used empirical analyses to prove that digitally inclusive finance has a positive spatial spillover effect on agricultural quality development. In terms of ecological livability, [34] constructed a rural ecological livability index system using the aspects of natural environment livability, artificial environment livability, and social environment livability and found that digitally inclusive finance can significantly improve the level of rural ecological livability, which and can be achieved through the effect of agricultural technology progress and urbanization. [35] showed that digitally inclusive finance can support farmers in planting green agricultural products with higher premiums and reduce the intensity of agricultural carbon emissions through channels such as improving the level of agricultural mechanization, optimizing the structure of agricultural cultivation, and expanding the scale of operation. In terms of rural civilization, [36] argued that digitally inclusive finance continuously records the credit activities of poverty-stricken people in an accurate, timely, and multi-dimensional way through digital credit collection, which helps farmers and low-income groups in poverty-stricken areas to establish a concept of credit and build and consolidate the concept of honesty and integrity in traditional culture. [37] argued that digitally inclusive finance reduces the burden on capital-constrained households, mitigates the problem of information asymmetry, and improves the educational attainment of low-income groups in terms of financial access. In terms of effective governance, [38] argued that cracking the “financial exclusion” and “threshold effect” of digital inclusive finance in China’s rural society can help innovate and improve the modernization and transformation of China’s rural governance model. [39] found that digitally inclusive finance can better help rural governance from the perspective of rural land contract disputes. In terms of livelihood affluence, [40] used an empirical analysis to determine that the development of digitally inclusive finance can effectively reduce the urban-rural income gap, while increasing Internet penetration can increase the inhibitory effect of digitally inclusive finance on the urban-rural income gap. [41] found that digitally inclusive finance empowers rural residents to increase their income through micro-mechanisms, such as easing the formal credit constraints of rural households, improving the production and operation of rural households, and raising the income level of rural households, thus reducing the urban-rural income gap. Based on the above analysis, this study proposes the following hypothesis:

Hypothesis 2.1: Digitally inclusive finance can effectively contribute to the realization of the dimensions of rural revitalization.

### 2.3 Digitally inclusive finance, rural revitalization, and rural consumption

The dynamic development of digitally inclusive finance has had a far-reaching impact on financial constraints in rural areas, providing more accessible and convenient channels for credit funds required by agribusinesses and farm households, which boosts the development of the rural economy and the income of residents. This triggers the overall level of consumption and improves its structure. Existing literature mainly includes the following paths: First, digitally inclusive finance has taken advantage of its own technology to expand the coverage of inclusive finance, the depth of use in the region, and the digitization of financial products for the majority of rural areas, improving the experience of financial services. Simultaneously digitally inclusive finance has supported the construction of infrastructure in rural areas [42,43], enhanced agricultural production, and mitigated agricultural risk management of agriculture-related enterprises, promoting the development and revitalization of the countryside. Second, with the revitalization of the countryside, rural areas’ economies further develop with the support of digitally inclusive finance, which further optimizes the digital infrastructure of the countryside [44]. Digitally inclusive finance not only provides a good financing environment for the development of agro-related enterprises, but also stimulates the entrepreneurial enthusiasm of rural residents, which steadily increases their income [45,46]. Third, along with the sustained growth of the economy in rural areas and improvement of the residents’ income level, the residents’ consumption ability also improves, which further optimizes the consumption structure of rural areas. Consequently, the said consumption structure will be of high-quality and become more diversified, which will promote the development of the overall consumer market. Based on the above analysis, we propose the following hypothesis:

Hypothesis 3: Rural revitalization mediates the relationship between digitally inclusive finance and rural residents’ consumption.

**Fig 1:**
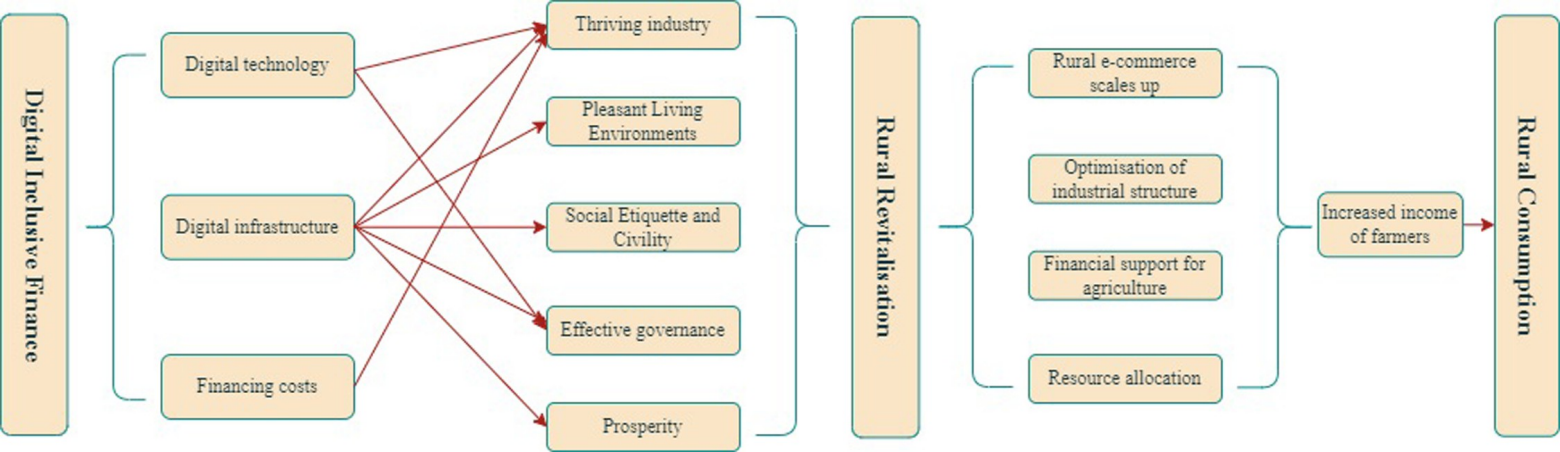
Mechanism analysis diagram.

Fig 1 shows a schematic representation of the mechanisms of digitally inclusive finance, rural revitalization, and rural consumption.

Extant research is laden with the following shortcomings. First, research on digitally inclusive finance and rural revitalization is mainly based on a linear perspective and does not consider the impact of time lag and the possible non-linear impact. Second, existing studies mainly study the direct impact of digitally inclusive finance on rural revitalization and do not consider whether rural revitalization has a mediating role in the relationship between digitally inclusive finance and rural residents’ consumption. Therefore, this study attempts to fill these research gaps.

## 3. Model setting and data description

### 3.1 Basic model setting

This study conducted empirical analyses based on panel data from 30 provinces and cities in China from 2011 to 2021 and constructs a model as follows (1). Considering the differences in the impact of digitally inclusive finance on different dimensions of rural revitalization, we constructed models for each dimension as follows [(2) to (6)].


$$C{\text{ons}}_{it}=\alpha +\beta_{1}Finid_{it}+\beta_{2}\;Rure_{it}+\sum \beta_{i}Controls_{it}+\mu_{it}+\varepsilon_{it}$$ (1)

$$Ti_{it}=\alpha +\beta_{1}Finid_{it}+\sum \beta_{i}Controls_{it}+\mu_{it}+\varepsilon_{it}$$ (2)

$$Ple_{it}=\alpha +\beta_{1}Finid_{it}+\sum \beta_{i}Controls_{it}+\mu_{it}+\varepsilon_{it}$$ (3)

$$Sec_{it}=\alpha +\beta_{1}Finid_{it}+\sum \beta_{i}Controls_{it}+\mu_{it}+\varepsilon_{it}$$ (4)

$$Eg_{it}=\alpha +\beta_{1}Finid_{it}+\sum \beta_{i}Controls_{it}+\mu_{it}+\varepsilon_{it}$$ (5)

$$Pr_{it}=\alpha +\beta_{1}Finid_{it}+\sum \beta_{i}\;Controls_{it}+\mu_{it}+\varepsilon_{it}$$ (6)

### 3.2 Mediating effects modelling

Combined with the above analysis, this study argued that digitally inclusive finance affects rural residents’ consumption, whereas rural revitalization mediates the impact of digitally inclusive finance on rural residents’ consumption. The specific mediation model was as follows (7)–(9): Considering that the rural revitalization indicator contains five dimensions, the mediating role of each dimension was further analyzed. Consequently, the following models were constructed [(10)–(14)].


$$C{\text{ons}}_{it}=\alpha +\beta_{1}Finid_{it}+\sum \beta_{i}Controls_{it}+\mu_{it}+\varepsilon_{it}$$ (7)

$$Rure_{it}=\alpha +\beta_{1}Finid_{it}+\sum \beta_{i}Controls_{it}+\mu_{it}+\varepsilon_{it}$$ (8)

$$C{\text{ons}}_{it}=\alpha +\beta_{1}Finid_{it}+\beta_{2}\;Rure_{it}+\sum \beta_{i}Controls_{it}+\mu_{it}+\varepsilon_{it}$$ (9)

$$C{\text{ons}}_{it}=\alpha +\beta_{1}Finid_{it}+\beta_{2}\;Ti_{it}+\sum \beta_{i}Controls_{it}+\mu_{it}+\varepsilon_{it}$$ (10)

$$C{\text{ons}}_{it}=\alpha +\beta_{1}Finid_{it}+\beta_{2}\;Ple_{it}+\sum \beta_{i}Controls_{it}+\mu_{it}+\varepsilon_{it}$$ (11)

$$C{\text{ons}}_{it}=\alpha +\beta_{1}Finid_{it}+\beta_{2}\;Sec_{it}+\sum \beta_{i}Controls_{it}+\mu_{it}+\varepsilon_{it}$$ (12)

$$C{\text{ons}}_{it}=\alpha +\beta_{1}Finid_{it}+\beta_{2}{Eg}_{it}+\sum \beta_{i}Controls_{it}+\mu_{it}+\varepsilon_{it}$$ (13)

$$C{\text{ons}}_{it}=\alpha +\beta_{1}Finid_{it}+\beta_{2}\;Pr_{it}+\sum \beta_{i}Controls_{it}+\mu_{it}+\varepsilon_{it}$$ (14)

here Cons represents the consumption of rural residents, Finid stands for digitally inclusive finance, Rure denotes rural revitalization, Control signifies the control variables, *α* is a constant term, *μ*_*it*_ is an individual fixed effect, and *ε*_*it*_ is an error term.

### 3.3 Threshold effect modelling

This study draws on Hansen’s (2000) threshold regression model and [47] model construction approach to construct the model as follows [(15)–(16)]:

$$\begin{array}{l} C{\text{ons}}_{it}=\alpha_{1}\;Rure_{it}*I\left(Rure_{it}\leq \theta_{1}\right)+\alpha_{2}\;Rure_{it}*I\left(\theta_{1}<\;Rure_{it}\leq \theta_{2}\right)\\ +\alpha_{3}\;Rure_{it}*I\left(Rure_{it}>\theta_{2}\right)+\beta \;Control_{it}+\varepsilon_{it} \end{array}$$ (15)

$$\begin{array}{l} C{\text{ons}}_{it}=\alpha_{1}Finid_{it}*I\left(Rure_{it}\leq \eta_{1}\right)+\alpha_{2}\;Rure_{it}*I\left(\eta_{1}<\;Rure_{it}\leq \eta_{2}\right)\\ +\alpha_{3}\;Rure_{it}*I\left(Rure_{it}>\eta_{2}\right)+\beta \;Control_{it}+\varepsilon_{it} \end{array}$$ (16)

where *θ*_1_, *θ*_2_⋯*θ*_*n*_ is the threshold for n levels, *Control*_*it*_ is a control variable, *Rure*_*it*_ is a threshold variable, *I*(•) is a dummy variable, and *μ*_*it*_ is a random disturbance term.

### 3.4 Variable selection

#### 3.4.1 Description of variables

This study selected rural residents’ per-capita consumption expenditure as the core explanatory variable to reflect changes in rural consumption. Digitally inclusive finance and rural revitalization were selected as explanatory variables. To guarantee the accuracy of the empirical results, financial support for agriculture, rural old-age dependency ratio, rural juvenile dependency ratio, level of opening up to the outside world, urbanization rate, industrial structure, consumer price index of rural residents, and level of economic development were selected as control variables (Table 1).

**Table 1:** Variable definition.

Variable name	Variable meaning	Construction method
cons	Consumption Expenditure per rural inhabitant	Consumption expenditure per rural resident treated in logarithmic terms
Finid	Digitally Inclusive Finance	From the Peking University Digital Inclusive Finance Index
Rure	Rural Revitalization	Measured using the entropy weight method
pub	Financial support for agriculture	Logarithmic treatment of financial expenditures on agriculture, forestry, and water as a percentage of total regional financial expenditures
Oldde	Rural old-age dependency ratio	Logarithmic treatment of the rural elderly population as a proportion of the labor force population
Chide	Rural child dependency ratio	Rural minors as a percentage of the labor force in logarithmic terms
Open	Trade openness	Total foreign trade imports and exports as a percentage of regional GDP
Urban	Urbanization rate	Urban population as a percentage of total regional population
Indus	Industrial structure	Measured by the ratio of the tertiary sector to the secondary sector

Fiscal support for agriculture signifies funds used to support rural development, involving all aspects of agriculture. In other words, not only improving rural infrastructure but also supporting the comprehensive development of agriculture, and generally promoting the stable development of agricultural and rural economies, which in turn promotes the growth of farmers’ income and demand [48].

Increases or decreases in both rural old-and young-age dependency ratios correspond to potential household cost expenditures for rural households. To better cope with old-age needs and the education of the next generation, there may be a reduction in consumption to increase savings and cope with future needs for money. Therefore, these two indicators impact rural residents’ consumption [49].

Level of openness to the outside world refers to the degree of communication between rural areas and domestic and foreign markets. A higher level of openness to the outside world means that rural areas can participate more extensively in international trade and external economic ties, which helps rural residents obtain more consumption choices, reduce consumption costs, and improve the quality and diversity of their consumption [50].

With the acceleration of urbanization in rural areas, rural residents are more easily exposed to urban consumption concepts, styles, and markets. Urbanization also enhances employment opportunities and opportunities to increase income, thus improving rural residents’ consumption capacity [51]. Therefore, urbanization rate significantly impacts rural residents’ consumption.

The transformation of the rural economy from traditional agriculture to modern agriculture, rural industry, and services not only increases employment opportunities and income levels in rural areas, but also provides a more diversified supply of goods. Therefore, the industrial structure may affect the consumption level and structure of rural residents [52].

The rural consumer price index directly reflects the changes in goods and services related to the daily lives of rural residents, and a sustained rise in this index will seriously affect residents’ daily lives and reduce consumption demand [53].

#### 3.4.2 Measurement of the level of rural revitalization

Rural revitalization is China’s development strategy and an inevitable requirement for achieving common prosperity; thus, the selection of rural revitalization indicators must fully reflect many aspects of development in rural areas. We referred to the methods of [54,55] and selected 29 basic indicators to quantify the level of rural revitalization using five dimensions: industrial prosperity, ecological livability, civilized rural culture, effective governance, and affluent life. The specific indicators are listed in Table 2.

**Table 2:** Indicator system of rural revitalization.

Dimensions	Basic indicators	Indicators to measure	Attribute
Thriving industry (Ti)	Grain production capacity (tones/thousand hectares)	Total food production/Total arable area under crops	positive
	Value added of primary industry (billions of dollars)	Value added of primary industry	positive
	Agricultural labor productivity (%)	Value added of primary sector/Total rural population	positive
	Land productivity (%)	Value added of primary sector/Crop sown area	positive
	Rural electricity consumption per capita (billion kWh/person)	Total rural electricity consumption/Total rural population	positive
	Mechanized power per acre (million kilowatts/thousand hectares)	Total power of agricultural machinery/Total arable area under crops	positive
	Fertilizer application per unit area (tones/thousand hectares)	Total fertilizer application/Total sown area of crops	negative
	Intensity of pesticide use (tones/thousand hectares)	Total pesticide applications/Total sown area of crops	negative
	Intensity of use of agricultural films (tones/thousand hectares)	Total agricultural plastic film use/Total sown area of crops	negative
	Fertilizer application per unit area (tones/thousand hectares)	Total fertilizer application/Total sown area of crops	negative
	Intensity of pesticide use (tones/thousand hectares)	Total pesticide applications/Total sown area of crops	negative
	Intensity of use of agricultural films (tones/thousand hectares)	Total agricultural plastic film use/Total sown area of crops	negative
Pleasant Living Environments (Ple)	Carbon emissions from agriculture (tones)	Carbon emissions from agriculture [56]	negative
	Forest cover (%)	Forest area/Total land area	positive
	Rural water supply penetration (%)	Percentage of administrative villages with centralized water supply	positive
	Rural gas penetration (%)	Ratio of population using gas in the countryside to total population at the end of the reporting period	positive
	Length of roads within villages (kilometers)	Length of roads within villages (kilometers)	positive
	Rural cable radio and television coverage (%)	Number of rural cable radio and television subscribers/total number of households	positive
	Village health technicians per 10,000 population (per cent)	Number of personnel in village health centers/Total rural population	positive
Social Etiquette and Civility (Sec)	Number of rural cultural centers (units)	Number of rural cultural centers	positive
	Population coverage of rural television programmers (%)	Rural television programmer population/Total rural population	positive
	Rural per capita years of schooling (years)	Percentage of workers with no education*1+Percentage of workers with primary school education*6+Percentage of workers with lower secondary school education*9+Percentage of workers with upper secondary school education*12+ Percentage of workers with post- secondary school education and above*16	positive
	Expenditure on education, culture, and recreation (%)	Expenditure on education, culture, and recreation of rural residents/Total expenditure of rural residents	positive
Effective governance (Eg)	Land treatment area (thousands of hectares)	Soil erosion control area +Flooded area	positive
	Area affected by crops (thousands of hectares)	Crops affected (thousands of hectares)	negative
	Length of drainage ditches (kilometers)	Drainage pipe trench length	positive
	Prevalence of sanitary latrines (%)	Number of sanitary latrines/Number of rural toilets	positive
Prosperity (Pr)	Rural-urban income balance (%)	Per capita disposable income of rural residents/Per capita disposable income of urban residents	positive
	Rural Engel’s coefficient (%)	Total food expenditure/Total consumption expenditure	negative
	Residential floor space per capita (square meters per person)	Residential floor area/Rural population	positive
	Average year-end mobile phone ownership per 100 rural households (units)	Rural inhabitants’ ownership of mobile telephones at the end of the year/100 households	positive
	Average year-end car ownership per 100 rural households (cars)	Average year-end car ownership per 100 rural households	positive

## 4. Empirical analysis

### 4.1 Data selection and processing

This study mainly selected the panel data of 30 provinces, autonomous regions, and municipalities directly under the central government of China, excluding Tibet, Hong Kong, Macao, and Taiwan, for the period of 2011–2021. The data on digitally inclusive finance are from the Digital Finance Research Centre of Peking University, while the data on other indicators are from the China Statistical Yearbook, China Rural Statistical Yearbook, China Urban and Rural Construction Statistical Yearbook, and the Wind Database. For missing individual data, the trend replacement method was used as a supplement. The descriptive statistics of the data are presented in Table 3.

**Table 3:** Descriptive statistics of the variables.

Variable	Obs	Mean	SD	Min	Max
Cons	330	9.214	0.382	8.258	10.211
Finid	330	2.315	1.033	0.183	4.590
Rure	330	0.562	0.092	0.299	0.783
Pub	330	2.711	0.3437	1.963	4.966
Oldde	330	2.905	0.380	1.953	3.824
Chide	330	3.269	0.384	1.834	4.076
Open	330	5.533	0.226	2.310	4.080
Urban	330	0.596	0.121	0.350	0.896
Indus	330	1.342	0.732	0.527	5.244
CPI	330	4.629	0.014	4.602	4.680

### 4.2 Measurement of rural revitalization index

Based on the calculation of 29 specific indicators according to five dimensions of rural revitalization, and using the entropy weighting method to calculate the rural revitalization development index [57,58], we found that the rural revitalization index of provinces and cities increased in 2021 compared with that in 2011. Shandong Province had the highest rural revitalization index (0.783), while Xinjiang had the lowest (0.523), with a mean value of 0.665. Guizhou showed the highest increase of 0.348, whereas Shanghai showed the lowest increase of 0.040. In terms of regional differences, the rural revitalization index of the eastern region was generally higher than that of the central region, and that of the central region was higher than that of the western region, with municipalities in the direct province of China having a certain impact due to their higher urbanization rate, smaller rural population, and smaller area. The results are summarized in Table 4.

**Table 4:** Index of rural revitalization.

Region	2011	2012–2015	2017	2018	2019	2020	2021
Beijing	0.555	………….	0.623	0.641	0.655	0.647	0.671
Tianjin	0.528	………….	0.652	0.649	0.660	0.658	0.703
Hebei	0.512	………….	0.631	0.651	0.680	0.700	0.733
Shanxi	0.438	………….	0.544	0.559	0.563	0.574	0.625
Inner Mongolia	0.407	………….	0.547	0.577	0.609	0.621	0.658
Liaoning	0.459	………….	0.579	0.578	0.609	0.595	0.640
Jilin	0.508	………….	0.576	0.581	0.608	0.621	0.666
Heilongjiang	0.489	………….	0.600	0.593	0.628	0.631	0.673
Shanghai	0.516	………….	0.593	0.601	0.601	0.583	0.556
Jiangsu	0.586	………….	0.709	0.710	0.727	0.729	0.742
Zhejiang	0.578	………….	0.692	0.700	0.713	0.724	0.737
Anhui	0.410	………….	0.556	0.589	0.613	0.626	0.668
Fujian	0.525	………….	0.641	0.661	0.691	0.695	0.726
Jiangxi	0.522	………….	0.625	0.654	0.665	0.681	0.704
Shandong	0.547	………….	0.713	0.721	0.739	0.765	0.783
Henan	0.444	………….	0.565	0.592	0.621	0.649	0.665
Hubei	0.464	………….	0.603	0.651	0.669	0.678	0.722
Hunan	0.423	………….	0.614	0.630	0.647	0.666	0.711
Guangdong	0.477	………….	0.596	0.626	0.659	0.682	0.718
Guangxi	0.412	………….	0.576	0.603	0.628	0.637	0.677
Hainan	0.366	………….	0.540	0.587	0.601	0.609	0.638
Chongqing	0.453	………….	0.541	0.565	0.585	0.604	0.624
Sichuan	0.455	………….	0.630	0.654	0.685	0.710	0.729
Guizhou	0.299	………….	0.542	0.569	0.602	0.618	0.647
Yunnan	0.375	………….	0.552	0.584	0.607	0.631	0.655
Shaanxi	0.466	………….	0.552	0.581	0.602	0.630	0.641
Gansu	0.329	………….	0.476	0.506	0.531	0.541	0.559
Qinghai	0.367	………….	0.509	0.519	0.547	0.563	0.571
Ningxia	0.407	………….	0.517	0.535	0.555	0.570	0.585
Xinjiang	0.335	…………	0.430	0.447	0.475	0.499	0.523

### 4.3 Basic regression and mediation effects analysis

This study first explored the relationship between digitally inclusive finance, rural revitalization, and rural residents’ consumption using an ordinary panel model. Since the LM and MLE tests strongly rejected the original hypothesis, and the p-value of the Hausman test was 0.000, which also strongly rejects the original hypothesis, this study chose the fixed-effects model for analysis.

In Table 5, Columns (1)–(4) test the impact of digitally inclusive finance on rural residents’ consumption and the mediating role of rural revitalization. Column (1) shows that digitally inclusive finance is significant at the 1% significance level for rural residents’ consumption, indicating that the development of digitally inclusive finance has a positive effect on rural residents’ consumption. This indicates that the advancement of digitally inclusive finance in the breadth of coverage, depth of use, and degree of digitization helps exert an income-generating effect, driving rural residents to increase their income and pursue higher levels of consumption demand. Column (2) indicates that the impact of digitally inclusive finance on rural revitalization is significant at the 1% significance level, with a coefficient of 0.025, thus verifying that Hypothesis 2 is valid; that is, digitally inclusive finance enhances rural residents’ consumption. Column (3) shows that the impact of rural revitalization on rural residents’ consumption is significant at the 1% significance level, with a coefficient of 2.015, thus verifying that Hypothesis 1 is valid; that is, rural revitalization promotes rural residents’ consumption. Column (4) shows that the effects of digitally inclusive finance and rural revitalization on rural residents’ consumption are also significant at the 1% significance level, with coefficients of 0.162 and 1.267, respectively, verifying that Hypothesis 3 is valid. This indicates that the development of digitally inclusive finance can bring about the redistribution of financial resources for rural development and promote the industrial development of the countryside, sustainable development of ecology, effectiveness of rural governance, and affluence of rural residents’ lives and alleviate the liquidity constraints of rural residents and enhance the consumption expenditure of rural residents.

**Table 5:** Results of basic regression and mediation effect analysis.

	(1) Cons	(2) Rure	(3) Cons	(4) Cons
Finid	0.194*** (9.34)	0.025***(3.07)		0.162***(8.76)
Rure			2.015***(5.06)	1.267*** (4.35)
Pub	-0.002(-0.23)	-0.002(-0.44)	0.012(0.75)	-0.001(-0.04)
Oldde	0.014(1.37)	-0.008* (-1.70)	0.068***(3.37)	0.024*(2.07)
Chide	0.007(0.65)	-0.001(-0.21)	-0.051*(-1.99)	0.009(0.91)
Open	0.021* (1.71)	0.003(0.66)	0.015(1.43)	0.017*(2.10)
Urban	1.941***(5.52)	0.988*** (6.67)	1.904***(3.34)	0.690(1.61)
Indus	0.105** (2.61)	0.015(0.85)	0.252***(4.71)	0.086*** (2.81)
CPI	0.083(0.25)	0.067(0.58)	-1.802***(-5.96)	-0.003(-0.01)
_Cons	7.014***(4.85)	-0.386(-0.79)	14.881***(10.69)	7.504***(6.33)
N	330	330	330	330

Note: ***, **, and * indicate significance at the 1%, 5%, and 10% levels, respectively.

We also examined the difference in the impact of digitally inclusive finance on the different dimensions of rural revitalization (Table 6). The results show that the difference in the impact of different dimensions of digitally inclusive finance on rural revitalization have a certain degree of significance, which verifies Hypothesis 2.1.

**Table 6:** Analysis results of different dimensions of rural revitalization.

	Ti	Ple	Sec	Eg	Pr
Finid	0.025***(3.11)	0.016**(2.47)	0.025**(4.22)	0.014*(1.72)	0.083***(10.96)
Pub	-0.006(-0.61)	-0.006(-0.82)	0.006*(0.91)	0.005(0.55)	-0.004(-0.46)
Oldde	0.011(1.15)	-0.006(-0.73)	-0.013*(-1.83)	0.009(0.89)	-0.002(-0.19)
Chide	-0.010(-0.92)	0.008(0.84)	0.013(1.57)	-0.016*(-1.41)	0.044***(4.16)
Open	-0.001(-0.09)	0.018*** (3.43)	0.005(1.12)	0.010(1.61)	-0.020***(-3.40)
Urban	-0.032(-0.24))	0.599***(5.40)	0.818***(8.29)	0.906***(6.69)	0.501***(4.00)
Indus	0.020(1.33)	-0.002(-0.15)	0.023**(2.01)	-0.009(-0.58)	0.006(0.39)
CPI	0.179(0.92)	0.449*** (2.73)	-0.225(-1.54)	0.712***(3.54)	0.241(1.30)
_cons	-0.683(-0.77)	-2.119***(-2.81)	0.787(1.17)	-3.598***(-3.91)	-1.349(-1.59)
N	330	330	330	330	330

Note: ***, **, and * indicate significance at the 1%, 5%, and 10% levels, respectively.

The mediation effect test for the different dimensions of rural revitalization is shown in Table 7. Columns (1)–(5) in Table 7 show the mediation effects of the five dimensions of rural revitalization on rural residents’ consumption through digitally inclusive finance. Among them, industrial prosperity was significant at the 10% level, rural civilization and affluent life were significant at the 1% level. However, the mediating effects of ecological livability and effective governance were not significant. This indicates that the impact of digitally inclusive finance on rural residents’ consumption through rural revitalization varies across dimensions, and the development of digitally inclusive finance mainly promotes the development of rural industries, improvement of rural civilization, and affluence of rural residents’ lives through the reallocation of financial resources. Overall, it eases the liquidity constraints of rural residents and increases rural residents’ consumption expenditure.

**Table 7:** Analysis of the mediating effect of the five dimensions of rural revitalization.

	(1) Cons	(2) Cons	(3) Cons	(4) Cons	(5) Cons
Finid	0.190*** (17.04)	0.193***(17.26)	0.175***(16.82)	0.194***(17.43)	0.170***(13.23)
Ti	0.148*(1.81)				
Ple		0.092(0.95)			
Sec			0.751***(7.53)		
Eg				0.035(0.44)	
Pr					0.287***(3.40)
Pub	-0.002(-0.13)	-0.002(-0.15)	-0.007(-0.61)	-0.003(-0.21)	-0.001(-0.10)
Oldde	0.013(0.92)	0.015(1.08)	0.024*(1.94)	0.014(1.02)	0.015(1.10)
Chide	0.009(0.57)	0.007(0.42)	-0.003(-0.18)	0.008(0.50)	-0.005(-0.34)
Open	0.021**(2.44)	0.019**(2.19)	0.017** (2.15)	0.020**(2.37)	0.027***(3.08)
Urban	1.946*** (10.64)	1.886***(9.79)	1.327***(7.09)	1.910***(9.67)	1.798***(9.70)
Indus	0.102*** (4.85)	0.105***(5.00)	0.088***(4.53)	0.105***(4.99)	0.103***(5.00)
CPI	0.056(0.21)	0.041(0.15)	0.252(1.00)	0.058(0.21)	0.013(0.05)
_cons	7.115*** (5.73)	7.209***(5.71)	6.423***(5.61)	7.139*** (5.58)	7.401***(6.02)
N	330	330	330	330	330

Note: ***, **, and * indicate significance at the 1%, 5%, and 10% levels, respectively.

### 4.4 Robustness checks

Considering the impact of extreme values of the sample of explanatory variables, we shrunk the explanatory variables by 1% [59] to ensure the robustness of the results. The regression results are shown in Table 8. The results show that digitally inclusive finance and rural revitalization have a facilitating effect on the consumption of rural residents, while the development of digitally inclusive finance can promote rural revitalization, thus indicating that rural revitalization has a mediating effect on the impact of digitally inclusive finance on rural residents’ consumption. This confirmed the robustness of the study’s results.

**Table 8:** Robustness checks results.

	(1)	(2)	(3)	(4)
	Cons	Rure	Cons	Cons
Finid	0.190***(9.18)	0.025***(3.07)	-	0.157***(9.60)
Rure	-	-	2.032***(5.13)	1.306***(4.79)
Pub	-0.003(-0.25)	-0.002(-0.44)	0.011(0.72)	-0.001(-0.06)
Oldde	0.014(1.29)	-0.008*(-1.70)	0.066***(3.32)	0.024*(2.02)
Chide	0.013(1.10)	-0.001(-0.21)	-0.044*(-1.91)	0.014(1.52)
Open	0.022*(1.82)	0.003(0.66)	0.016(1.48)	0.017**(2.28)
Urban	1.996***(6.17)	0.988***(6.67)	1.884***(3.22)	0.706(1.62)
Indus	0.106**(2.60)	0.015(0.85)	0.248***(5.23)	0.087***(2.85)
CPI	0.118(0.36)	0.067(0.58)	-1.716***(-5.95)	0.030(0.11)
_cons	6.810***(4.71)	-0.386(-0.79)	14.472***(10.99)	7.314***(6.18)
N	330	330	330	330

Note: ***, **, and * indicate significance at the 1%, 5%, and 10% levels, respectively.

### 4. 5 Heterogeneity analysis

Given that there are certain differences in economic development between Chinese provinces, the sample was divided into eastern, central, and western regions for the heterogeneity test. The test results are presented in Table 9, which shows that the east, central, and west are basically the same as the national results, mainly because of the relatively slow development of the countryside in the central and western regions, the serious exodus of the young population, the insufficient development of the industry, and the fact that the utilization of digitally inclusive finance is yet to be further improved. Therefore, it can be assumed that digitally inclusive finance has a promoting effect on rural residents’ consumption, while rural revitalization mediates the relationship between digital inclusive finance and rural residents’ consumption.

**Table 9:** Heterogeneity analysis.

Variable	(1) Eastern				(2) Central				(3) Western			
	Cons	Rure	Cons	Cons	Cons	Rure	Cons	Cons	Cons	Rure	Cons	Cons
Finid	0.175*** (6.07)	0.022*** (3.92)	-	0.139*** (5.88)	0.208*** (6.45)	0.024* (1.74)	-	0.176** (3.38)	0.180*** (4.20)	0.023** (2.05)	-	0.156*** (3.84)
rure	-	-	2.388*** (3.29)	1.575** (2.86)	-	-	1.682** (2.93)	1.383** (2.77)	-	-	1.281** (2.70)	1.017** (2.61)
Pub	-0.022 (-1.29)	-0.009 (-0.98)	0.014 (0.65)	-0.008 (-0.64)	0.008 (0.68)	0.002 (0.43)	0.012 (1.17)	0.005 (0.52)	-0.100 (-0.64)	0.095** (2.21)	-0.002 (-0.01)	-0.196 (-1.45)
Oldde	0.025 (1.02)	-0.019** (-2.20)	0.128*** (4.09)	0.055 (1.79)	0.078*** (3.71)	0.012 (1.05)	0.076** (2.99)	0.061** (2.73)	0.004 (0.23)	0.004 (0.53)	-0.006 (-0.35)	0.001 (0.00)
Chide	0.018 (0.96)	0.004 (0.53)	-0.042* (-2.17)	0.012 (0.99)	-0.017 (-0.69)	-0.006 (-0.50)	-0.059 (-1.78)	-0.008 (-0.29)	0.007 (0.27)	-0.027* (-1.98)	0.009(4.69)	0.034* (1.84)
Open	-0.003 (-0.20)	-0.005 (-1.00)	0.009 (0.85)	0.005 (0.39)	0.037* (1.95)	0.046*** (3.35)	-0.113* (-2.23)	-0.026 (-0.57)	0.049** (3.01)	0.007 (1.56)	0.059*** (4.69)	0.042** (3.07)
Urban	1.800*** (3.80)	1.077*** (9.92)	0.919 (1.03)	0.103 (0.20)	1.298 (1.57)	0.768** (2.66)	3.340*** (4.41)	0.235 (0.18)	2.972*** (4.30)	0.877*** (4.58)	3.991*** (4.67)	2.080** (2.33)
Indus	0.163* (2.23)	0.026** (2.35)	0.277** (3.20)	0.121** (2.84)	0.077* (2.16)	0.024 (1.63)	0.053 (0.99)	0.045 (1.01)	0.018 (0.37)	-0.014 (-0.79)	0.028 (0.70)	0.032 (0.82)
CPI	-0.651 (-1.44)	-0.018 (-0.11)	-2.203*** (-6.10)	-0.623 (-1.70)	0.367 (1.22)	-0.151 (-0.71)	-1.210 (-1.80)	0.575 (1.15)	0.409 (0.61)	0.290 (1.57)	-1.406** (-2.61)	0.114 (0.17)
_cons	10.394*** (5.38)	-0.085 (-0.12)	16.765***(10.19)	10.527*** (6.73)	5.982*** (4.22)	0.732 (0.79)	11.839***(3.82)	4.970** (2.44)	5.427* (1.83)	-1.497* (-1.81)	12.772***(4.69)	6.950** (2.30)
N	121	121	121	121	88	88	88	88	121	121	121	121

Note: ***, **, and * indicate significance at the 1%, 5%, and 10% levels, respectively.

### 4.6 Analysis of threshold effects

Before regressing the threshold variables, we determined whether there was a threshold effect and the number of thresholds using bootstrap sampling (bootstrap) of 300 regressions (Tables 10 and 11).

**Table 10:** Threshold effect self-sampling test results.

Independent variable	Threshold variables	Hypothesis testing	RSS	MSE	F-statistic	P-value
Rure	Rure	Single Threshold	0.944	0.003	32.180	0.013
		Double Threshold	0.905	0.003	13.730	0.187
		Triple Threshold	0.887	0.003	6.420	0.683
L1. Rure	Rure	Single Threshold	0.867	0.003	25.960	0.040
		Double Threshold	0.814	0.003	18.900	0.057
		Triple Threshold	0.796	0.003	6.710	0.747
Finid	Rure	Single Threshold	0.649	0.002	37.250	0.000
		Double Threshold	0.624	0.002	12.650	0.323
		Triple Threshold	0.605	0.002	9.850	0.737
L1.Finid	Rure	Single Threshold	0.57	0.002	43.090	0.010
		Double Threshold	0.519	0.002	30.760	0.010
		Triple Threshold	0.507	0.00	6.870	0.860

**Table 11:** Threshold estimates and confidence intervals.

Independent variable	Threshold variables	Threshold	Estimated value	95% confidence interval
Rure	Rure	First threshold	0.400	[0.388 0.404]
Finid	Rure	First threshold	0.552	[0.549 0.554]
L1.Rure	Rure	First threshold	0.552	[0.551 0.384]
		Second threshold	0.658	[0.649 0.659]
L1.Finid	Rure	First threshold	0.520	[0.508 0.522]
		Second threshold	0.584	[0.578 0.585]

#### 4.6.1 Threshold effect test for rural revitalization

When rural revitalization was both an independent variable and a threshold variable, the results showed that rural revitalization has a single threshold, with an F-statistic of 32.18, which is significant at the 5% level. The single threshold value for rural revitalization was 0.400, with a 95% confidence interval of [0.388–0.404]. Considering that the promotion of rural revitalization requires a process, it will not be completed immediately; therefore, there will be a dual-threshold effect for the lag phase of the rural revitalization process, wherein the second threshold value is 0.658, with a 95% confidence interval of [0.649 0.659].

#### 4.6.2 Threshold effect test for digital inclusive finance

When the independent variable was digitally inclusive finance, rural revitalization was significant at the 1% significance level in the single-threshold test. The single-threshold value was 0.5520, with a 95% confidence interval of [0.549 0.554]. Considering that the development of digitally inclusive finance also requires a process, there was a dual-threshold effect for the lagging period of digitally inclusive finance, wherein the single threshold value was 0.520, with a 95% confidence interval of [0.508 0.522], and the second threshold value was 0.584, with a 95% confidence interval of [0.578 0.585]. After surpassing the first threshold value, as digitally inclusive finance develops and improves, the effect is further enhanced.

The parameters were estimated for the sample based on the values of each threshold indicator, and the results of the threshold regression are shown in Table 12.

**Table 12:** Threshold regression estimates.

	(1) Cons	(2) Cons	(3) Cons	(4) Cons
Independent variable	Rure	L. Rure	Finid	L. Finid
Threshold variables	Rure	Rure	Rure	Rure
Rure (*Rure* ≤ *θ*_1_)	2.306***(6.46)			
Rure (*Rure* > *θ*_1_)	2.037***(5.79)			
Rure (*Rure* ≤ *θ*_1_)		1.463***(4.51)		
Rure (*θ*_1_ < *Rure* ≤ *θ*_2_)		1.576***(4.76)		
Rure (*Rure* ≥ *θ*_2_)		1.647***(5.01)		
Finid (*Rure* ≤ *η*_1_)			0.167***(9.12)	
Finid (*Rure* > *η*_2_)			0.187***(10.25)	
Finid (*Rure* ≤ *η*_1_)				0.130***(8.19)
Finid (*η*_1_ < *Rure* ≤ η_2_)				0.159***(9.36)
Finid (*Rure* > η_2_)				0.178***(11.07)
Oldde	0.062***(3.78)	0.042**(2.58)	0.001(1.03)	-0.002(-0.18)
Chide	-0.050*(-2.15)	-0.061***(-3.62)	0.001(1.14)	-0.036***(-3.51)
Open	0.004(0.44)	0.011(1.14)	0.005(0.52)	0.025**(2.46)
Urban	2.009***(4.09)	2.166***(4.17)	1.855***(10.60)	1.895***(6.10)
Indus	0.257***(5.07)	0.235***(4.83)	0.099***(4.88)	0.086***(3.04)
CPI	-1.977***(-6.65)	-1.895 ***(-5.13)	-0.445(-1.52)	-1.404***(-4.25)
Pub	0.012(0.76)	0.011(0.59)	-0.001(-0.08)	-0.005(-0.34)
_cons	8.469***(28.13)	15.589***(9.14)	9.493***(7.43)	14.307***(9.69)
R-squared	0.765	0.748	0.853	0.839
F	435.81	311.47	400.59	667.10

Note: ***, **, and * indicate significance at the 1%, 5%, and 10% levels, respectively.

The regression results for rural revitalization and rural residents’ consumption were analyzed. Column (1) of Table 12 shows that when rural revitalization was less than or equal to 0.400, there was a significant positive impact on rural residents’ consumption. However, when rural revitalization was less than or equal to 0.400, its stimulating effect on consumption was significant. Further, when rural revitalization was greater than 0.400, its stimulating effect on consumption diminished, presenting an inverted “U”-shaped relationship. Column (2) in Table 12 shows the results of the lagged one period of rural revitalization regression: when rural revitalization was less than or equal to 0.552, there was a significant positive impact on residents’ consumption (the coefficient was 1.463). When rural revitalization was between 0.552 to 0.658, the positive impact on rural residents’ consumption became stronger. Further, when rural revitalization was greater than 0.658, the significant positive impact on rural consumption was further strengthened. Therefore, there is a nonlinear positive relationship between rural revitalization and rural consumption with a lag. Additionally, the control variables exhibited significant correlations with rural consumption. Urbanization rate and industrial structure positively affect consumption, implying that modern living aspirations and increased income drive consumption. Aging populations are increasing their spending on health, leisure, and driving. Conversely, the child dependency ratio negatively impacts consumption, as families save on future education expenses. Furthermore, the Consumer Price Index (CPI) negatively affects consumption because rising prices reduce rural residents’ purchasing and spending power.

#### 4.6.3 Analysis of the regression results for digitally inclusive finance and rural residents’ consumption

Columns (3) and (4) of Table 12 show the results for digitally inclusive finance in the current and lagged periods. The results in column (3) indicate that when rural revitalization was less than or equal to 0.552, there was a significant positive impact of digitally inclusive finance on rural residents’ consumption; when rural revitalization was greater than 0.552, there was a significant positive impact on rural residents’ consumption and the coefficient further increased. Therefore, there is a nonlinear positive relationship between digitally inclusive finance and rural residents’ consumption in the current period; that is, the higher the level of digitally inclusive finance development, the greater is the effect on rural residents’ consumption. Column (4) of Table 12 presents lagged one period of digitally inclusive finance regression results, indicating that when rural revitalization was less than or equal to 0.520, digitally inclusive finance had a significant positive impact on rural residents’ consumption. Further, when rural revitalization was between 0.520 and 0.584, the positive impact of digitally inclusive finance on rural residents’ consumption was strengthened, with the coefficient rising to 0.159. Finally, when rural revitalization was greater than 0.584, the positive impact on rural residents’ consumption increased. Essentially, when revitalization was greater than 0.584, there was a significant positive impact on rural residents’ consumption, with the coefficient increasing to 0.178. Therefore, there is a nonlinear positive relationship between digitally inclusive finance and rural residents’ consumption, with a lag period.

## 5. Conclusions of the study and recommendations for countermeasures

This study empirically investigates the impact of digitally inclusive finance on rural residents’ consumption and the mediating and threshold effects of rural revitalization based on panel data from 30 provinces and cities in China from 2011 to 2021. Heterogeneity analysis was conducted based on the regional differences in eastern, central, and western China. The empirical results show that: (1) there is a significant positive promotion effect of digitally inclusive finance on rural residents’ consumption, while rural revitalization shows a significant mediating effect; however, there are differences in the mediating effects based on the dimensions of rural revitalization. The mediating effects of ecological livability and effective governance are not significant. (2) The impact of rural revitalization on rural residents’ consumption exhibits a single-threshold effect, whereas the effect of rural revitalization lagged by one period on rural residents’ consumption shows a dual-threshold effect, with an overall increasing trend. (3) The impact of digitally inclusive finance on rural residents’ consumption exhibits a single-threshold effect of rural revitalization, demonstrating a clear promoting effect on rural residents’ consumption. The effect of digitally inclusive finance lagged by one period on rural residents’ consumption shows a dual-threshold effect of rural revitalization, with an overall trend of increasing gradient.

Based on these conclusions, this study puts forward the following two suggestions:

First, the government should prioritize the improvement of rural information sharing and digital infrastructure to boost digital financing and rural consumption. This implies increasing network investment for comprehensive coverage, especially in remote areas, to support digital finance. In addition, establishing information sharing mechanisms can address financing difficulties, reduce costs, and support rural economic development. Furthermore, regulating digital finance products is crucial for preventing wasteful development and excessive consumption in rural areas.

Second, to foster rural development, the government should boost policy support and resource allocation, while igniting the entrepreneurial spirit of rural residents. This involves promoting modern agricultural techniques, enhancing product quality, and increasing incomes. Diversifying rural industries to create jobs and income is crucial. Additionally, upgrading rural infrastructure, such as transportation and utilities, is essential for bridging the urban-rural divide. Prioritizing sustainable energy sources improves living standards and environmental sustainability. Furthermore, investing in rural education, healthcare, and social services enhances the well-being of rural residents. Developing rural tourism and cultural sectors enriches rural life and its appeal.

This study investigated the correlation between digitally inclusive finance, rural revitalization, and rural consumption based on existing theories. However, there are areas for improvement. First, the selection of rural revitalization indicators relies mainly on statistical yearbook data, which limits comprehensive insights. Future research should conduct practical studies to gather more extensive data and enhance the measurement of rural revitalization. Additionally, while this study employs a panel model for empirical analysis, future studies could explore alternative empirical methods such as spatial econometric or systematic GMM models for a deeper analysis of the relationships between digital finance, rural revitalization, and rural consumption.

## Supporting information

S1 File Dataset. Data.
(RAR)
